# Modulation of biochemical constituents, nutritional status, growth and flowering of *Brunfelsia grandiflora* via supplying diverse silicon sources

**DOI:** 10.1038/s41598-025-21117-z

**Published:** 2025-10-01

**Authors:** Iman M. El-Sayed, Eman Z. Othman, Mohammed Hewidy, Hani S. Saudy, Rasha A. M. El-Ziat

**Affiliations:** 1 Ornamental Plants and Woody Trees Department, National Research Centre (NRC), Dokki, Cairo Egypt; 2 Ornamental Horticulture Department, Faculty of Agriculture, Cairo University, Giza, Egypt; 3 Horticulture Department, Faculty of Agriculture, Ain Shams University, 68-Hadayek Shoubra, 11241 Cairo, Egypt; 4 Agronomy Department, Faculty of Agriculture, Ain Shams University, 68-Hadayek Shoubra, 11241 Cairo, Egypt

**Keywords:** Diatomaceous silicon, Healthy flowers, Nanotechnology, Ornamentals, Secondary metabolites, Physiology, Plant sciences

## Abstract

Recently, several countries have been using silicon (Si) fertilization in agricultural plants. The application of Si has a beneficial role on plant growth via affecting the cellular metabolites and physiological events. However, there are little findings that interpret the impact of different Si sources on physiological and agronomical traits of *Brunfelsia grandiflora*. Along the two seasons of 2022 and 2023, this study examined the impact of different Si sources (diatomite (DM), which comprises several elements and soluble SiO_2_ (86–89%), potassium silicate K_2_O_5_Si_2_ (PS), and silica-nanoparticles (SNP)) at different rates on biochemical constituents, nutritional status, growth and flowering of *B. grandiflora*. In addition to check treatment (no Si application), three levels of each of DM (2.5%, 5%, and 10%; DM2.5, DM5, and DM10, respectively), PS (1, 2 and 3 g L^–1^; PS1, PS2 and PS3, respectively), and SNP (100, 200, and 300 mg L^− 1^; SNP100, SNP200 and SNP300, respectively) were sprayed three times at four-week intervals, initiating 30 days after planting. The estimated data showed that DM and SNP as sources of Si had remarkable potential for ameliorating photosynthetic pigments, anthocyanin, nutrients content and secondary metabolites, hence the morphological and flowering traits of *B. grandiflora*. SNP had a positive impact comparable with DM or PS. Foliar application of SNP100 exhibited the maximum increases in plant pigments concentration with high healthy status and flower production. However, the supplementation of DM as a natural Si fertilizer should not be neglected where DM2.5 had acceptable growth and flowering potential compared to PS and untreated plants. Si supplementation, particularly with SNP and DM, improved morphological and floral traits by boosting pigment content (photosynthetic and anthocyanin), increasing phenolics and flavonoids level, and enhancing overall antioxidant capacity. The growers are advised to insert Si in fertilization programs of *B. grandiflora* to obtain high flower yield and quality. According to the availability, application of silica-nanoparticles at 100 mg L^− 1^ or diatomite 2.5% are costless and good practices to nourish *B. grandiflora* plants.

## Introduction

As a result of the increasing interest and desire to acquire ornamental plants for the aesthetic appearance they provide, scientific attempts have increased to solve the problems of trading these plants^1–3^. Researchers are interested in finding effective ways to improve the productivity and quality of ornamentals to extend their life spam^4–6^. For enhancing the productivity and quality, plants should receive the appropriate amounts and types of nutrients^7–11^. The inappropriate nutrients supply could expose the plants to stress hazards, suppressing growth and development^12–15^. In particular, nutritional stress resulting from nutrient deficiencies leads to physiological dysfunction due to oxidative damage, reducing crop yield and quality^16–19^.

The Solanaceae family includes the genus *Brunfelsia*, which provides several flowering plants^20^. About 50 species of shrubs and small trees can be found in Brazil. The leaves are simple and often oval^21^. *Brunfelsia* species are sold under “yesterday-today-tomorrow” and “lady of the night”; the phrase “Yesterday-Today-Tomorrow” refers to the blossom’s change from a deep purple color yesterday to a lavender color today before turning white tomorrow^22^. It is nocturnally performed and color-changing flowers from dark purple over mauve to white^23,24^. Thickets and light woodland are the typical habitats for these plants. Numerous *Brunfelsia* species contain toxic alkaloids and medicinal. For instance, *B. grandiflora* is the source of the most significant native remedies used to treat arthritis, rheumatism, and snake bites in the upper Amazon region^25^. According to the phytochemical analysis, steroids, flavonoids, tannins, and saponins were found in the methanol leaf extract^26^. However, terpenoids, anthraquinones, and cardiac glycosides were absent. DPPH activity of leaf extract and ascorbic acid standard was found. The studies found that this plant has a high level of antioxidant activity. Other *Brunfelsia* species including *B. calycina*and *B. grandiflora* have been cultivated for their ornamental properties for pot plants and a common garden due to their vast blue flowers and appealing aroma^21,22^.

Although micronutrients are required in small quantities, their abundance is absolutely essential for plants to complete their healthy growth and produce high-quality, marketable products^27–29^. In this respect, silicon (Si) is a crucial beneficial element for high-yield cultivation of ornamentals, and other economic crops^30–32^. Si deficiency causes suboptimal growth and induces stress, reducing photosynthetic efficiency and increasing susceptibility to pests and pathogenic diseases^33,34^. Contrariwise, the abundance of Si promotes the growth, making plants healthier by enhancing structural integrity and facilitating key physiological processes involved in cell development and differentiation^35^. This is because various plant species have differing abilities to absorb Si, largely determined by the presence and efficiency of specific Si transporters in their roots^36,37^. Furthermore, previous studies have shown that Si plays a crucial role in the tolerance of plants to heavy metal stress, primarily through mechanisms such as metal immobilization in the soil, compartmentalization within plants, and the upregulation of antioxidant defenses^38,39^. Similarly, Si is critical for mitigating biotic stress, as its deposition in plant tissues creates a physical barrier that deters pests and pathogens and primes the plant’s jasmonic acid and ethylene-mediated defense pathways^40,41^.

Utilization of natural sources of nutrients is considered a promising practice to enhance plant growth and yield with high quality^42–45^. Herein, diatomite de Mozambique is a naturally occurring sedimentary rock mostly made up of the fossilized remains of freshwater diatoms. Chemically, it comprises several elements and soluble SiO_2_ (86–89%) available to plants. Diatomite (DM) achieves a multifaceted positive influence on plant growth, flowering, and development. It showed a positive response for number, weight, and tonnage of melon fruits to diatomite application^46^. Studies on various flowering plants have shown that substrate amendment with DM can lead to earlier flowering, increased flower number, and prolonged bloom duration, attributed to the overall improvement in plant health and nutrient availability^47^.

Potassium silicate (PS) is a highly soluble potassium and Si source. It is used in agricultural production systems primarily as Si amendment source and uses a small amount of potassium (K) to improve the quality of flowers^48^. K-silicate is a source of highly soluble K and Si. PS does not volatile organic compounds^49^. The use of PS improved vegetative parameters and led to earlier flowering, increased flower number of *Tagetes patula*^50^. Application of PS increased carotenoids, total phenols, flavonoids, and anthocyanin and K content, while decreased malondialdehyde content at all salinity levels on *Cichorium intybus*^51^.

Fertilizers prepared in nano form exhibited beneficial role for efficiently utilization of nutrients and induction of plants to be more tolerant to adverse conditions^52–54^. Herein, silica-nanoparticles (SNP) are ultra-small particles made primarily of silicon dioxide (SiO_2_). It significantly affects plant growth, protection of plants, and physiology. SNP can dramatically enhance the processes of water absorption and nutrient supply, positively regular photosynthesis and gas exchange, and active metabolic processes, improving the antioxidant defense system and nitrogen metabolism^55,56^. Further, Using SNP reduces the harmful effects of stresses such as UV radiation, salinity, drought, metal toxicity, and biotic stress^57,58^. Additionally, SNP can act directly as nano-fertilization, nano pesticides, and nano–herbicides^59^.

Si was used on bushy growing plants such as *Glycyrrhiza uralensis* and *Glycyrrhiza inflata*^60^, and *Thunbergia erecta*^61^. Practically, *B. grandiflora* have not received any studies regarding the importance of Si and its various forms in regulating metabolic processes and stimulating growth and flowering. Therefore, the current research hypothesized that the different types of Si fertilizers could influence the biochemical constituents, nutritional status, growth and flowering of plants. To prove this assumption, the effects of three Si sources at three rates each on *B. grandiflora* were investigated.

## Materials and methods

### Location and plant materials

The present research was conducted under open-field conditions at the experimental site of the Ornamental Horticulture Department, Faculty of Agriculture, Cairo University, Giza, Egypt. The experiment spanned the growing seasons from March to September in 2022 and 2023 seasons. The region experiences a Mediterranean climate characterized by arid summers and negligible precipitation. Throughout the primary growth period, the mean ambient temperature, relative humidity and mean daily solar radiation were 25.0 °C, 52.4%, 28.7 MJ m⁻² day⁻¹, respectively. The study tested the impact of different silicon sources (diatomite DM, potassium silicate, PS, and silica-nanoparticles, SNP) on biochemical constituents, nutritional status, growth and flowering of *B. grandiflora*. The seedlings of *B. grandiflora* were obtained from a commercial farm in Giza, Egypt. The seedlings were cultivated in 40 cm lengths in plastic pots (40 cm diameter). Each pot involved one seedling, and the soil mixture medium was peat moss, perlite, and compost 1:1:1 by volume (totally, 25 kg).

### The applied treatments and design

The treatments were arranged in randomized complete block design with three replicates. DM was added to the soil medium a week before transplanting at three levels (2.5%, 5%, and 10%, denoted DM2.5, DM5 and DM10, respectively). PS (K_2_SiO_3_) was applied at three concentrations (1, 2, and 3 g L^− 1^ abbreviated as PS1, PS2 and PS3, respectively), as a foliar spray. SNP were sprayed at three concentrations (100, 200, and 300 mg **L**^**-1**^ coded as SNP100, SNP200 and SNP300, respectively), applied as leaf application. The control plants (CK) were treated with distilled water. For PS and SNP, the plants received the spray solutions three times until the runoff point, the first spray applied 30 days after planting, then in four-week intervals, “Tween 20” was applied at one ml L^− 1^ as a surfactant substance.

### Specification of SNP

**Fig. 1:**
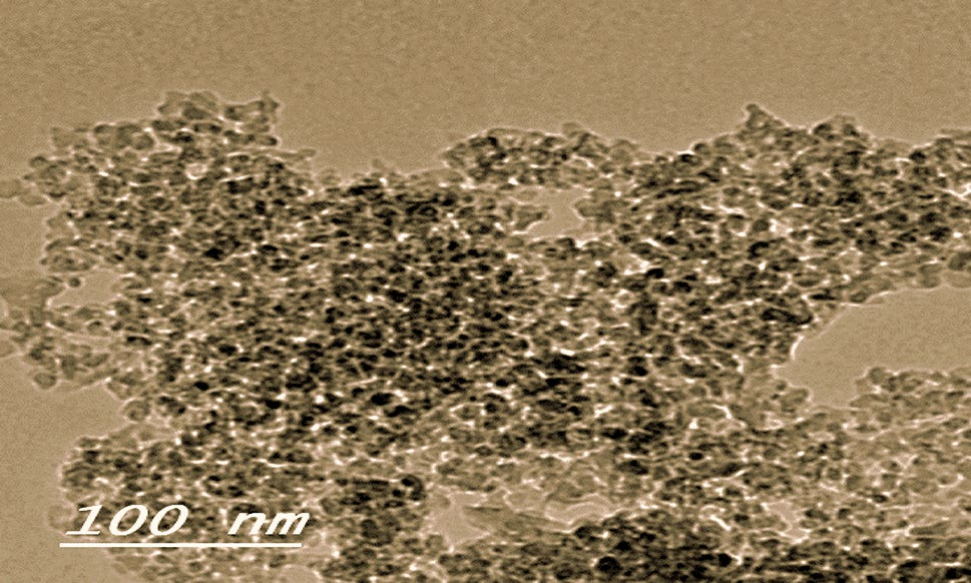
Scanning electron microscopy image of silica-nanoparticles at 100 nm.

The Electron NRC microscopy unit was used to characterize SNP that were obtained from Sigma Cor. in the USA. According to Zafar et al. 2016^62^, SNP are suspended in uniformly dispersed and distilled water. The specification of SNP is illustrated in Table 1; Fig. 1.

**Table 1:** Specification of used silica oxide nanoparticles.

Property	Specification	Test method
Phase	Silica gel	XRD
Particle size	<50 nm	TEM
Surface area	>200 m^2^ g^–1^	BET (P/Po: up to 0.35)

### Assessments

#### Plant pigments

The photosynthetic pigments, i.e. chlorophyll a, chlorophyll b, and carotenoids were determined as explained by Saric et al. 1967^63^ in mature leaves. According to the method of Fuleki and Francis 1968^64^, an ethanol hydrochloric acid solution (15 ml 1.5 N HCl + 85 ml ethanol 95%) was used for extraction to determine the anthocyanin pigment in fully open flower at full bloom; four months after beginning of the treatments.

#### Biochemical constituents

Using fresh leaves sample, flavonoids content^65^ and total phenolics content^66^ were assessed. Total antioxidant activity (DPPH) in terms of hydrogen-donating or radical-scavenging ability, using the stable radical method described by Brand-Williams et al. 1995^67^ was estimated.

#### Nutrients content

Nitrogen (N) content of dried shoots was performed by the modified Kjeldahl method^68^. Phosphorus (P) content was determined by the method of the ammonium molybdate in dry shoots^69^. The extract for determination of potassium (K) content was prepared from a digested solution in the dry shoot according to Chapman and Pratt 1961^70^.

Application inductively coupled plasma optical emission spectrometry (ICP-OES) was used to determine Si content utilizing the microwave-assisted digestion technique^71^. The concentrations of magnesium (Mg) and iron (Fe) were assessed by an atomic absorption spectrophotometer with air acetylene and fuel (PyeUnicam, model SP-1900, US) as explained by Cheng and Bray 1951^72^.

#### Plant growth and flowering

After 6 months from transplanting, plant height (measured from the base of plant to the tip of the plant by measuring tape), branches number plant^–1^, leaves number plant^–1^, leaf area (estimated as total leaf dry weight × disks area / disks dry weight), and flowers number plant^–1^ were recorded.

### Statistical analysis

The results were statistically analyzed by employing COSTATV-63 program^73^. One way analysis of variance (ANOVA) was used to evaluate the significance by Duncan’s new multiple-range tests at *p* < 0.05. Pearson correlation coefficients matrix between the studied traits expressed in heat map was prepared using excel program, and COSTATV-63 program was employed to estimate the significance test at *p* < 0.05, *p* < 0.01 and *p* < 0.001.

## Results

### Plant pigments

**Fig. 2:**
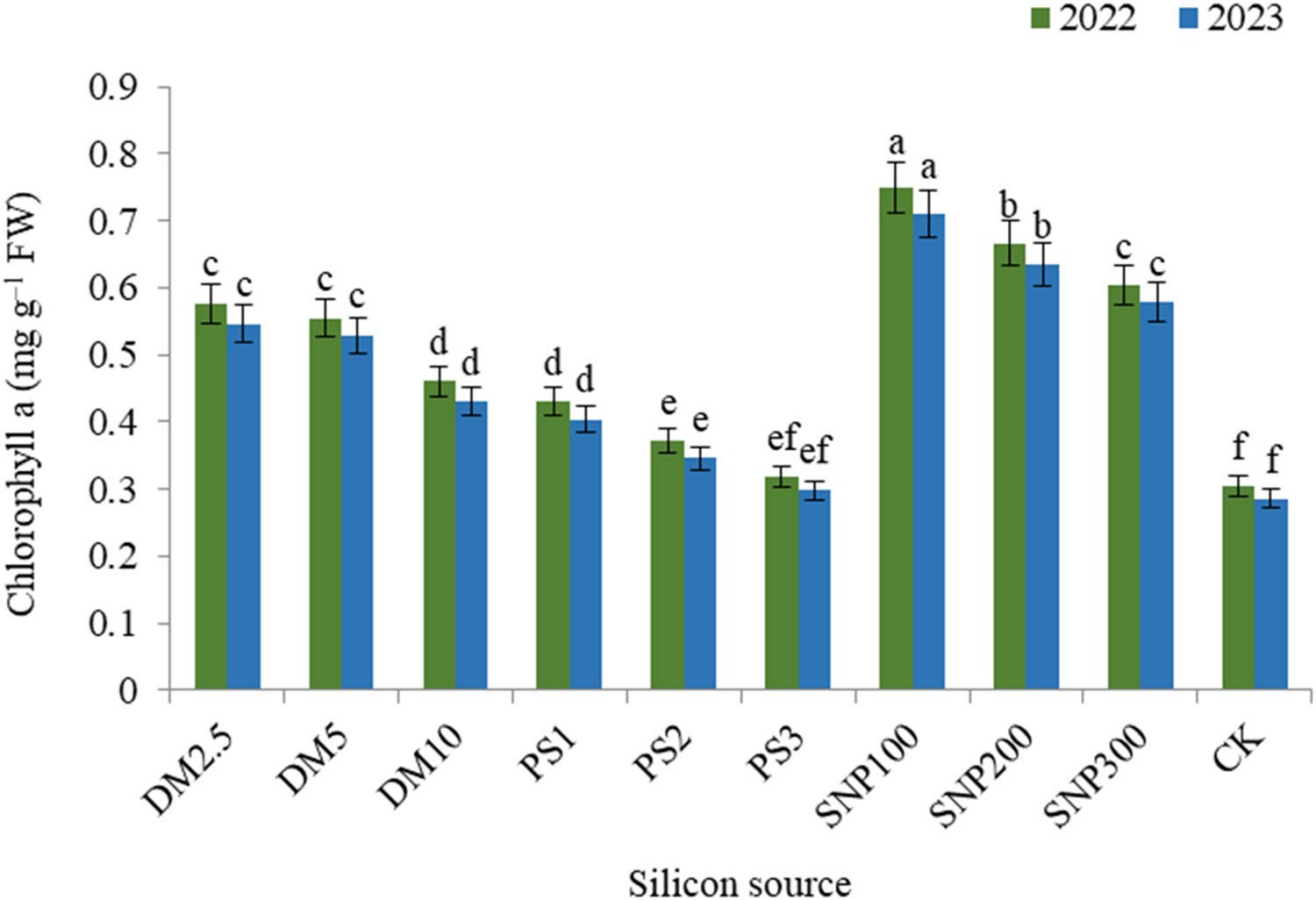
Effect of diatomite, potassium silicate and silica-nanoparticles on chlorophyll a content (mg g^–1^ FW) of *Brunfelsia grandiflora* in 2022 and 2023 seasons. DM2.5, DM5 and DM10 are application 2.5, 5 and 10% of diatomite, respectively; PS1, PS2 and PS3 are application of potassium silicate at a rate of 1, 2 and 3 g L^–1^, respectively; SNP100, SNP200 and SNP300 are application of silica-nanoparticles at a rate of 100, 200, and 300 mg L^− 1^, respectively; CK is check treatment. Values are means of 3 replicates ± SE; the same alphabets means that statistically non-significant by Duncan’s new multiple range test at *p* < 0.05.

**Fig. 3:**
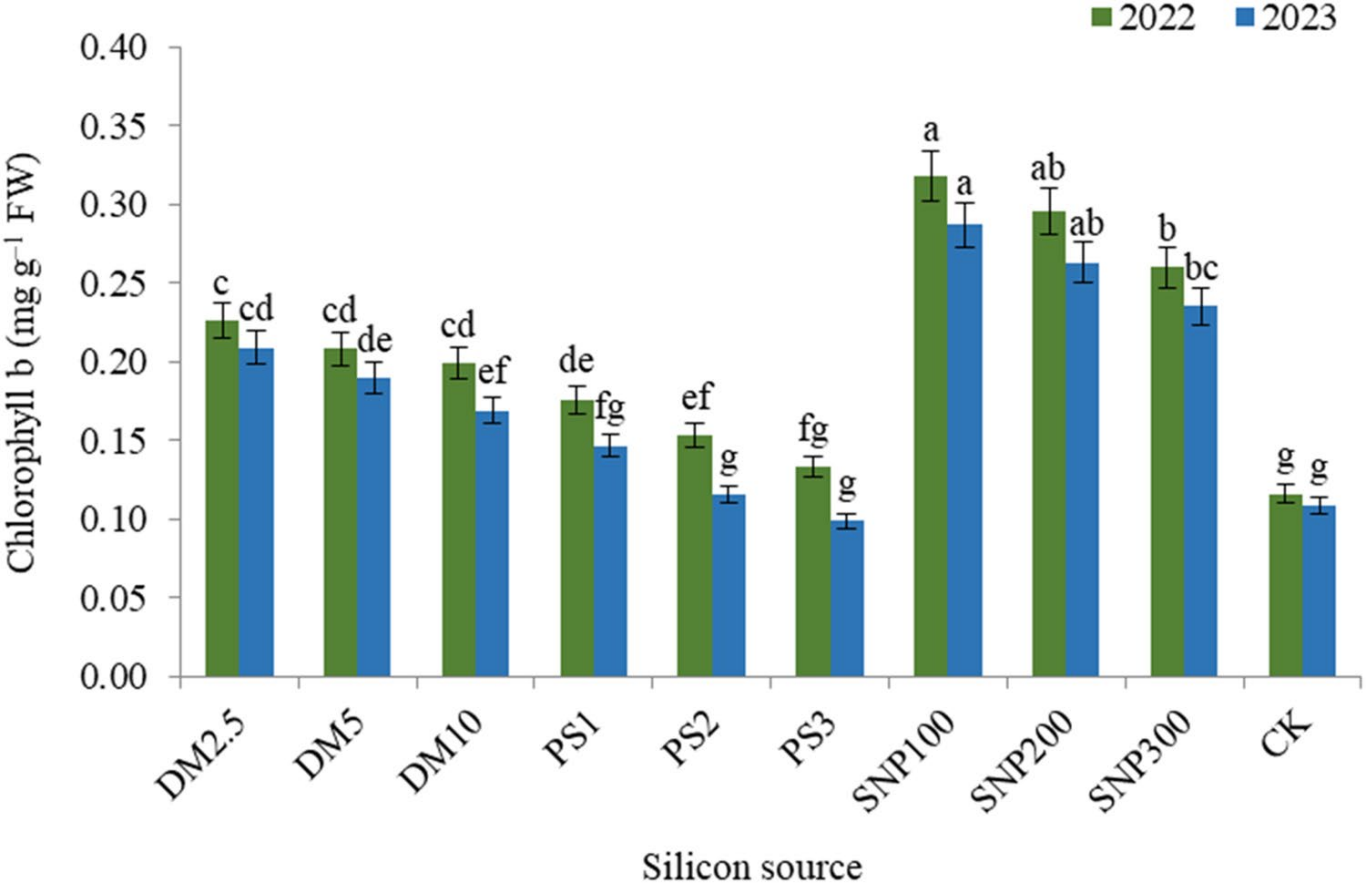
Effect of diatomite, potassium silicate and silica-nanoparticles on chlorophyll b content (mg g^–1^ FW) of *Brunfelsia grandiflora* in 2022 and 2023 seasons. DM2.5, DM5 and DM10 are application 2.5, 5 and 10% of diatomite, respectively; PS1, PS2 and PS3 are application of potassium silicate at a rate of 1, 2 and 3 g L^–1^, respectively; SNP100, SNP200 and SNP300 are application of silica-nanoparticles at a rate of 100, 200, and 300 mg L^− 1^, respectively; CK is check treatment. Values are means of 3 replicates ± SE; the same alphabets means that statistically non-significant by Duncan’s new multiple range test at *p* < 0.05.

**Fig. 4:**
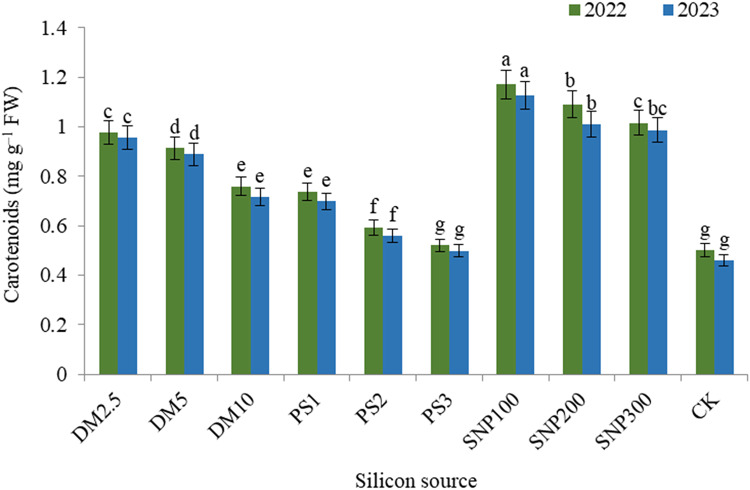
Effect of diatomite, potassium silicate and silica-nanoparticles on carotenoids content (mg g^–1^ FW) of *Brunfelsia grandiflora* in 2022 and 2023 seasons. DM2.5, DM5 and DM10 are application 2.5, 5 and 10% of diatomite, respectively; PS1, PS2 and PS3 are application of potassium silicate at a rate of 1, 2 and 3 g L^–1^, respectively; SNP100, SNP200 and SNP300 are application of silica-nanoparticles at a rate of 100, 200, and 300 mg L^− 1^, respectively; CK is check treatment. Values are means of 3 replicates ± SE; the same alphabets means that statistically non-significant by Duncan’s new multiple range test at *p* < 0.05.

**Fig. 5:**
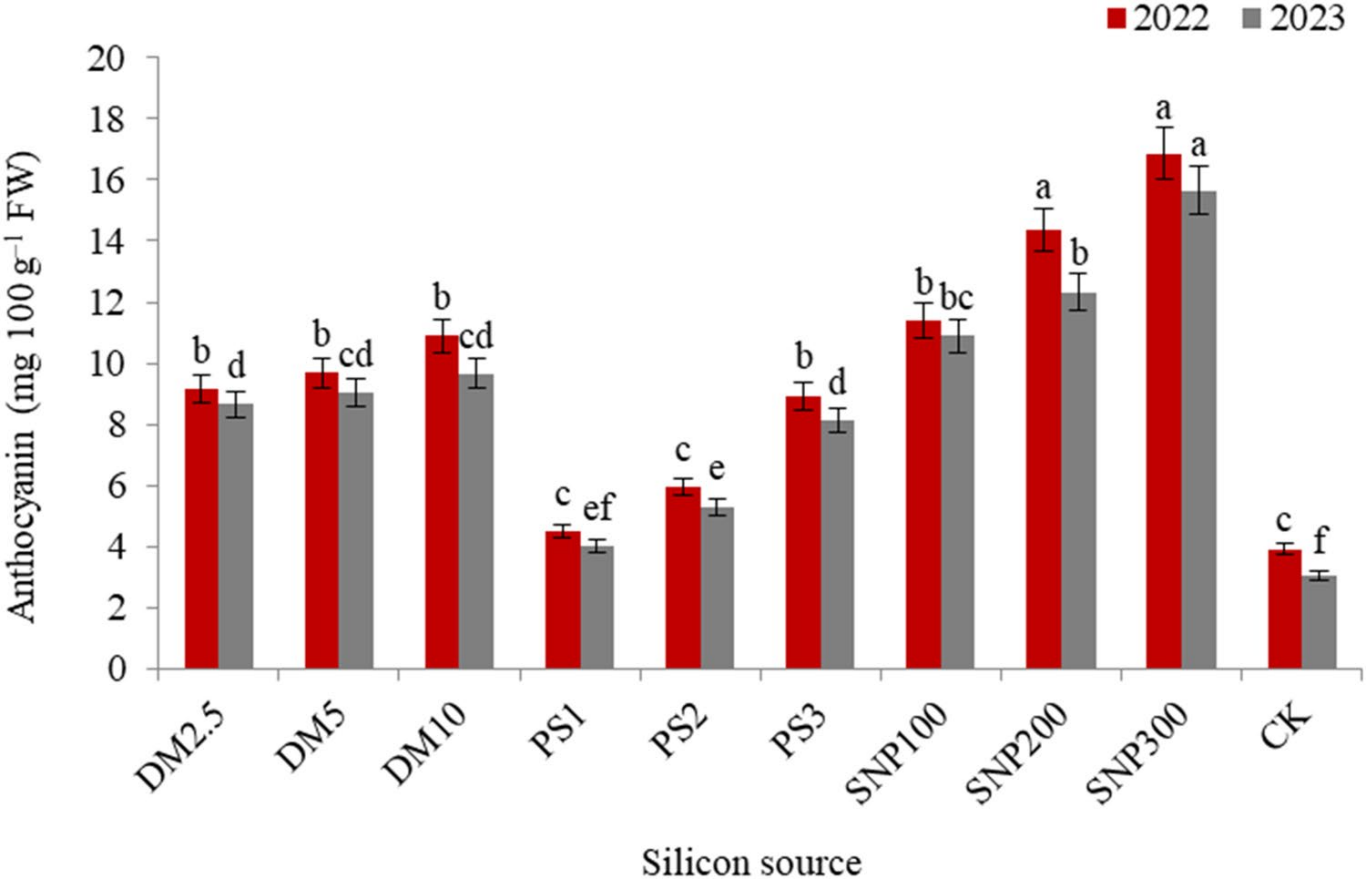
Effect of diatomite, potassium silicate and silica-nanoparticles on anthocyanin content (mg 100 g^–1^ FW) of *Brunfelsia grandiflora* in 2022 and 2023 seasons. DM2.5, DM5 and DM10 are application 2.5, 5 and 10% of diatomite, respectively; PS1, PS2 and PS3 are application of potassium silicate at a rate of 1, 2 and 3 g L^–1^, respectively; SNP100, SNP200 and SNP300 are application of silica-nanoparticles at a rate of 100, 200, and 300 mg L^− 1^, respectively; CK is check treatment. Values are means of 3 replicates ± SE; the same alphabets means that statistically non-significant by Duncan’s new multiple range test at *p* < 0.05.

The impacts of DM, PS and SNP on the concentration of plant pigments were significant. In this concern, data illustrated that the concentration of chlorophyll a (Fig. 2), chlorophyll b (Fig. 3), and carotenoids (Fig. 4) decreased and anthocyanin (Fig. 5) increased gradually with increasing the application rate of all silicon forms. For diatomite rates, application of DM2.5 outperformed DM10 in chlorophyll a as well as DM5 and DM10 in carotenoids in both seasons. Also, PK1 application showed greater values of chlorophyll a, chlorophyll b, and carotenoids that of PK2 or PK3 in both seasons. SNP100 increased chlorophyll a by 24.0 and 23.0%, chlorophyll b by 22.3 and 22.1%, and carotenoids by 15.2 and 14.1% in the first and second seasons, respectively, as compared to SNP300. Among the potassium sources overall, SNP100 was the potent treatment for improving all photosynthetic pigments in both seasons, significantly leveling SNP200 for chlorophyll b.

Concerning the anthocyanin pigment, SNP300 showed the highest values surpassed the other treatments in both seasons, except SNP200 in the first season. Compared to the check treatment, SNP300 increases the concentration of anthocyanin by about 4.32 and 5.2 times in 2022 and 2023 seasons, respectively.

### Biochemical constituents

**Fig. 6:**
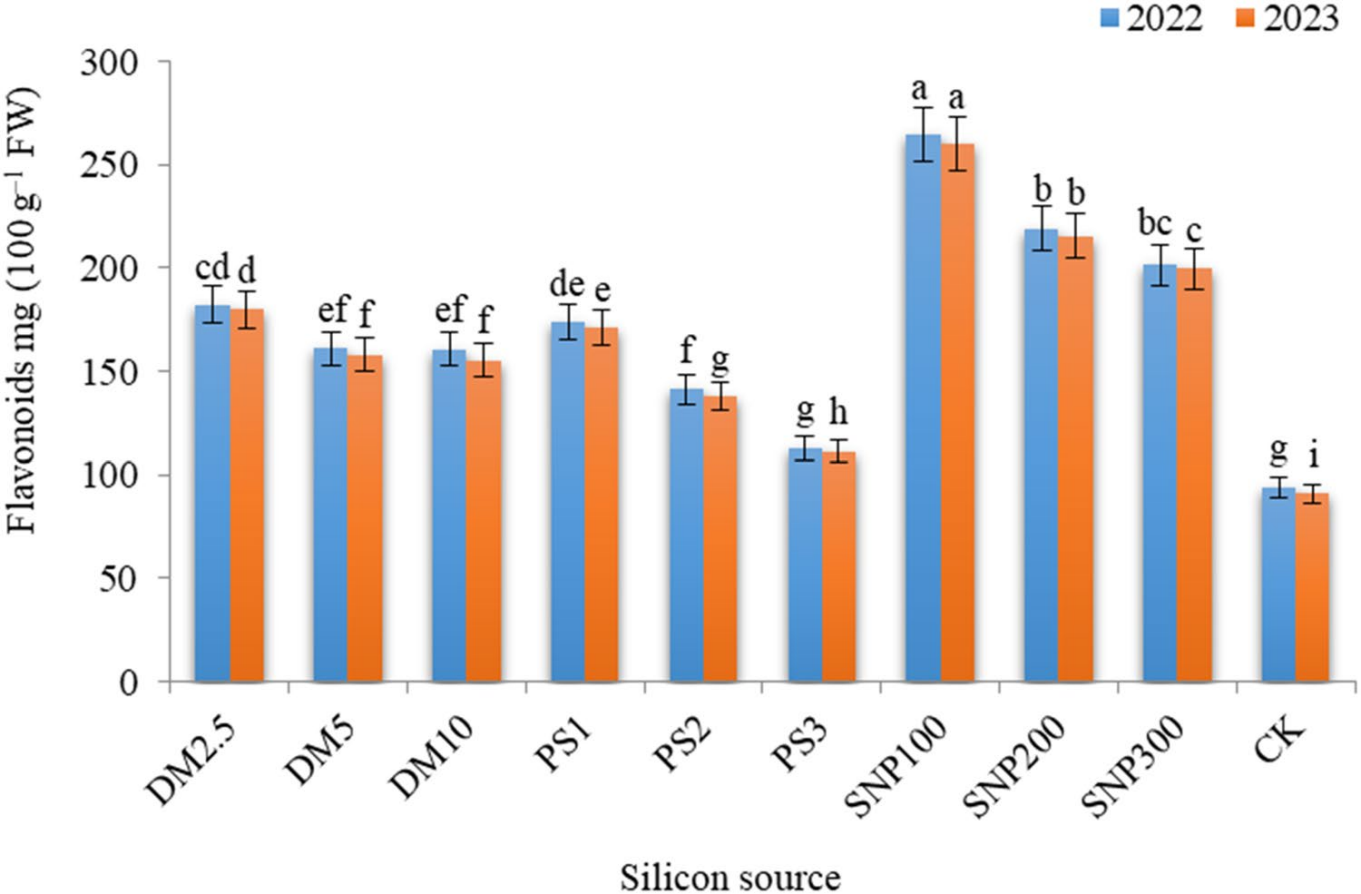
Effect of diatomite, potassium silicate and silica- nanoparticles on flavonoids content (mg 100 g^–1^ FW) of *Brunfelsia grandiflora* in 2022 and 2023 seasons. DM2.5, DM5 and DM10 are application 2.5, 5 and 10% of diatomite, respectively; PS1, PS2 and PS3 are application of potassium silicate at a rate of 1, 2 and 3 g L^–1^, respectively; SNP100, SNP200 and SNP300 are application of silica-nanoparticles at a rate of 100, 200, and 300 mg L^− 1^, respectively; CK is check treatment. Values are means of 3 replicates ± SE; the same alphabets means that statistically non-significant by Duncan’s new multiple range test at *p* < 0.05.

**Fig. 7:**
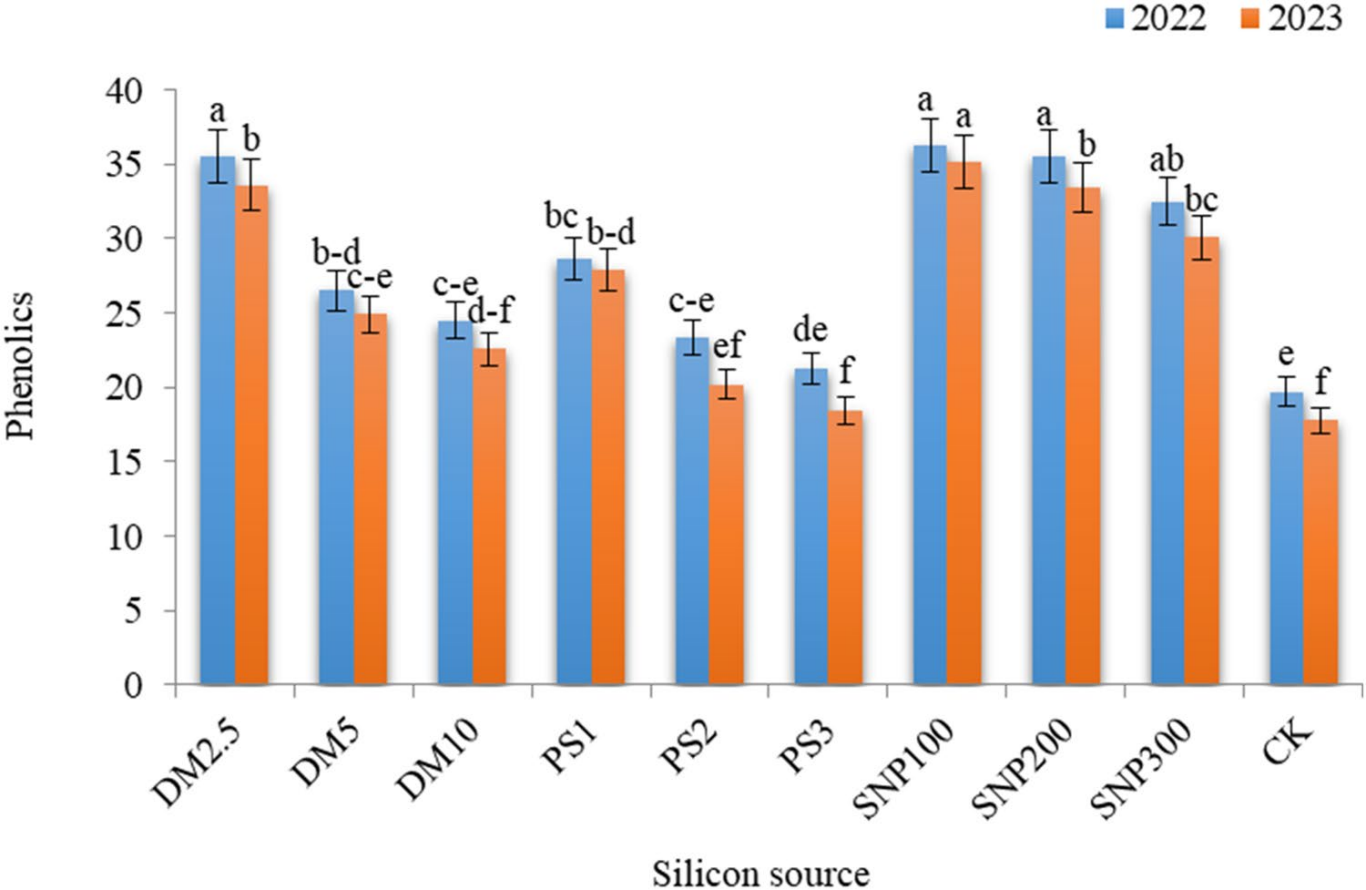
Effect of diatomite, potassium silicate and silica-nanoparticles on phenolics content (mg g^–1^ FW) of *Brunfelsia grandiflora* in 2022 and 2023 seasons. DM2.5, DM5 and DM10 are application 2.5, 5 and 10% of diatomite, respectively; PS1, PS2 and PS3 are application of potassium silicate at a rate of 1, 2 and 3 g L^–1^, respectively; SNP100, SNP200 and SNP300 are application of silica-nanoparticles at a rate of 100, 200, and 300 mg L^− 1^, respectively; CK is check treatment. Values are means of 3 replicates ± SE; the same alphabets means that statistically non-significant by Duncan’s new multiple range test at *p* < 0.05.

**Fig. 8:**
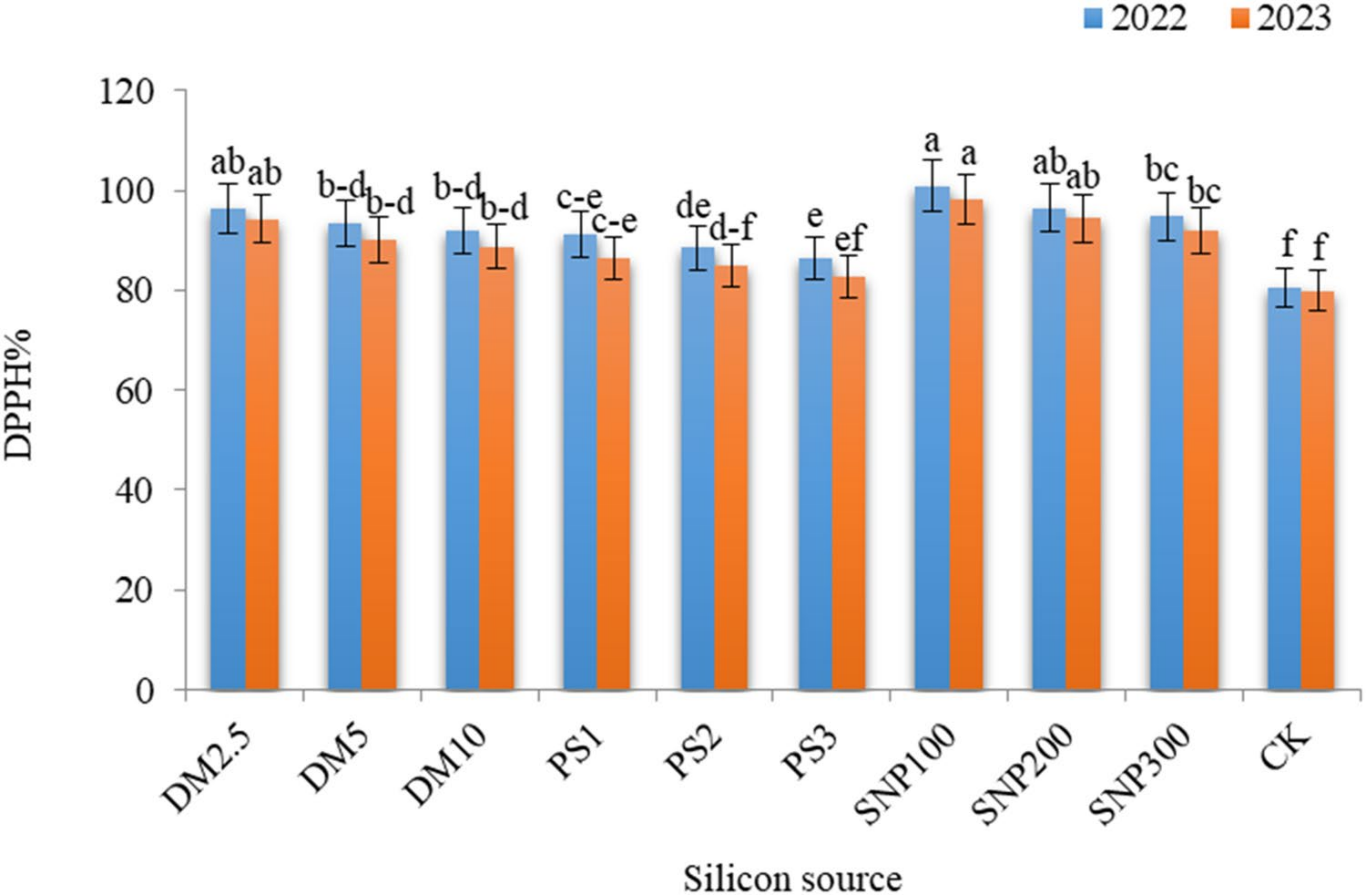
Effect of diatomite, potassium silicate and silica-nanoparticles on total antioxidant activity (DPPH) (%) of *Brunfelsia grandiflora* in 2022 and 2023 seasons. DM2.5, DM5 and DM10 are application 2.5, 5 and 10% of diatomite, respectively; PS1, PS2 and PS3 are application of potassium silicate at a rate of 1, 2 and 3 g L^–1^, respectively; SNP100, SNP200 and SNP300 are application of silica-nanoparticles at a rate of 100, 200, and 300 mg L^− 1^, respectively; CK is check treatment. Values are means of 3 replicates ± SE; the same alphabets means that statistically non-significant by Duncan’s new multiple range test at *p* < 0.05.

Remarkable influences of DM, PS, and SNP on the total flavonoids (Fig. 6), phenolics (Fig. 7), and DPPH (Fig. 8) in the leaves of *Brunfelsia grandiflora* were obtained in both seasons. SNP100 gave the maximum values of total flavonoids, phenolics and DPPH%, significantly equaling DM2.5, SNP200 and SNP300 for phenolics in the first season as well as DM2.5 and SNP200 for DPPH in both seasons. The increases in flavonoids, phenolics, and DPPH due to application of SNP100 were 2.81 and 2.85 folds, 1.84 and 1.97 folds and 1.25 and 1.23 folds, greater than the check treatment in the first and second seasons, respectively. It should be noted progressive decreases in all measured biochemical constituents with increasing the application rates of any potassium source. Thus, DM10, PK3 and SNP300 exhibited lower values of flavonoids, phenolics, and DPPH as compared DM2.5, PK1 and SNP100, respectively.

### Nutrients content

The rates of DM, PS, and SNP affected the concentration of N, P, K, Mg, Si and Fe in *Brunfelsia grandiflora* leaves. Data results in Table 2 showed that the lowest application rates of each potassium form concentrations were more effective for increasing nutrients content. Among the different forms of Si, SNP100 exhibited the highest efficiency for raising the leaf content of N, P, Mg, Si and Fe in both seasons. However, there were no significant differences between SNP100 and SNP200 or DM2.5 for Mg and SNP200 for Si in both seasons. On the other hand, PK1 was the efficient treatment for elevating K content surpassing the other treatments in both seasons.

**Table 2:** Effect of diatomite (DM), potassium silicate (PS), and silica-nanoparticles (SNP) on nutrients content of Brunfelsia grandiflora leaves in 2022 and 2023 seasons.

Variable	Nitrogen (%)	Phosphorus (%)	Potassium (%)	Magnesium (%)	Silicon (%)	Iron (ppm)
Season of 2022						
DM2.5	1.31b	0.543b	5.55ef	2.40a	2.35bc	194.7c
DM5	1.01 cd	0.379d	4.83 g	1.81bc	1.90 cd	155.1e
DM10	0.98d	0.352d	4.30 h	1.63 cd	1.88d	138.6f
PS1	1.28b	0.452c	8.06a	1.99b	1.91 cd	181.5d
PS2	0.87e	0.249e	7.70b	1.38de	1.85d	132.0f
PS3	0.85e	0.195ef	6.27c	1.25e	1.32e	88.3 g
SNP100	1.64a	0.789a	5.91d	2.45a	3.20a	290.4a
SNP200	1.31b	0.547b	5.37ef	2.13ab	2.98a	217.8b
SNP300	1.09c	0.542b	5.33f	2.08b	2.44b	198.7c
CK	0.84e	0.146s	4.12i	1.22e	0.74f	69.3 h
Season of 2023						
DM2.5	1.27b	0.476b	5.01de	2.31a	2.22b	190.5c
DM5	0.99e	0.349d	4.77e	1.76bc	1.79 cd	151.2e
DM10	0.94ef	0.337d	4.21f	1.60 cd	1.65d	130.4f
PS1	1.19 cd	0.409c	7.50a	1.94a-c	1.87c	175.1d
PS2	0.9 fg	0.223e	7.11b	1.30de	1.80 cd	126.5f
PS3	0.84gh	0.178e	6.14b	1.21de	1.28e	82.5 g
SNP100	1.43a	0.669a	5.72bc	2.35a	2.88a	276.3a
SNP200	1.25bc	0.513b	5.3 cd	2.09ab	2.75a	211.5b
SNP300	1.15d	0.503b	5.24c-e	2.01a-c	2.36b	188.5c
CK	0.78 h	0.119f	3.88f	1.17e	0.65f	65.8 h

DM2.5, DM5 and DM10 are application 2.5, 5 and 10% of diatomite, respectively; PS1, PS2 and PS3 are application of potassium silicate at a rate of 1, 2 and 3 g L^–1^, respectively; SNP100, SNP200 and SNP300 are application of silica-nanoparticles at a rate of 100, 200, and 300 mg L^− 1^, respectively; CK is check treatment. Values are means of 3 replicates ± SE; the same alphabets means that statistically non-significant by Duncan’s new multiple range test at *p* < 0.05.

### Plant growth and flowering

According to the results of Table 3, different silicon sources (DM, PS and SNP) significantly influenced growth and flowering yield of *Brunfelsia grandiflora*. All Si sources and their concentrations remarkably enhanced plant height, branches number plant^–1^, leave number plant^–1^, leaf area and flowers number plant^–1^ comparing to the check treatment in both seasons, except PS and its rates for branches number plant^–1^, which equaled the check treatment in this respect. SNP100 was the most effective practice for promoting plant growth and flowering traits in both season. However, there were no significant variations between SNP100 and each of SNP200 and SNP100 for enhancing branches number plant^–1^ and plant leaf area in both season, in addition to SNP100 and SNP200 for leave number plant^–1^, in the first season.

**Table 3:** Effect of diatomite (DM), potassium silicate (PS), and silica-nanoparticles (SNP) on growth and flowering of Brunfelsia grandiflora in 2022 and 2023 seasons.

Variable	Plant height (cm)	Branches number plant^–1^	Leaves number plant^–1^	leaf area (cm^2^)	Flowers number plant^–1^
Season of 2022					
DM2.5	137.5bc	15.5bc	305.0bc	46.34ab	149.0c
DM5	130.0 cd	15.0c	294.0b-d	45.33bc	137.0d
DM10	126.5de	14.0c	278.0c-e	44.66b-d	130.0e
PS1	125.0de	12.5 cd	270.0c-e	43.33b-d	120.0f
PS2	123.0de	11.8 cd	265.0de	42.50 cd	115.0f
PS3	119.5e	10.5d	243.0ef	42.00de	109.0 g
SNP100	149.8a	23.0a	393.0a	51.66a	173.0a
SNP200	144.7ab	20.0a	370.0a	49.00a	165.0b
SNP300	139.4b	19.5ab	325.0b	46.83ab	160.0b
CKT	98.0f	10.0d	225.0f	39.33e	65.0 h
Season of 2023					
DM2.5	128.2bc	14.0bc	287.0d	44.60a-c	142.0c
DM5	120.7 cd	13.5bc	276.0d	42.00bc	130.0d
DM10	117.2de	12.5 cd	258.0e	41.00b-d	123.0e
PS1	118.7de	11.0c-e	244.0f	40.00b-d	113.0f
PS2	113.7de	10.3c-e	232.0 fg	38.44 cd	108.0 fg
PS3	110.2e	9.0de	220.0 g	35.70 cd	102.0 g
SNP100	140.5a	21.5a	370.0a	49.20a	166.0a
SNP200	135.4ab	19.0a	347.0b	48.30ab	158.0b
SNP300	130.1b	18.0ab	312.0c	46.50a-c	153.0b
CK	88.7f	8.0e	202.0 h	32.00d	58.0 h

DM2.5, DM5 and DM10 are application 2.5, 5 and 10% of diatomite, respectively; PS1, PS2 and PS3 are application of potassium silicate at a rate of 1, 2 and 3 g L^–1^, respectively; SNP100, SNP200 and SNP300 are application of silica-nanoparticles at a rate of 100, 200, and 300 mg L^− 1^, respectively; CK is check treatment. Values are means of 3 replicates ± SE; the same alphabets means that statistically non-significant by Duncan’s new multiple range test at *p* < 0.05.

### Correlation analysis

**Fig. 9:**
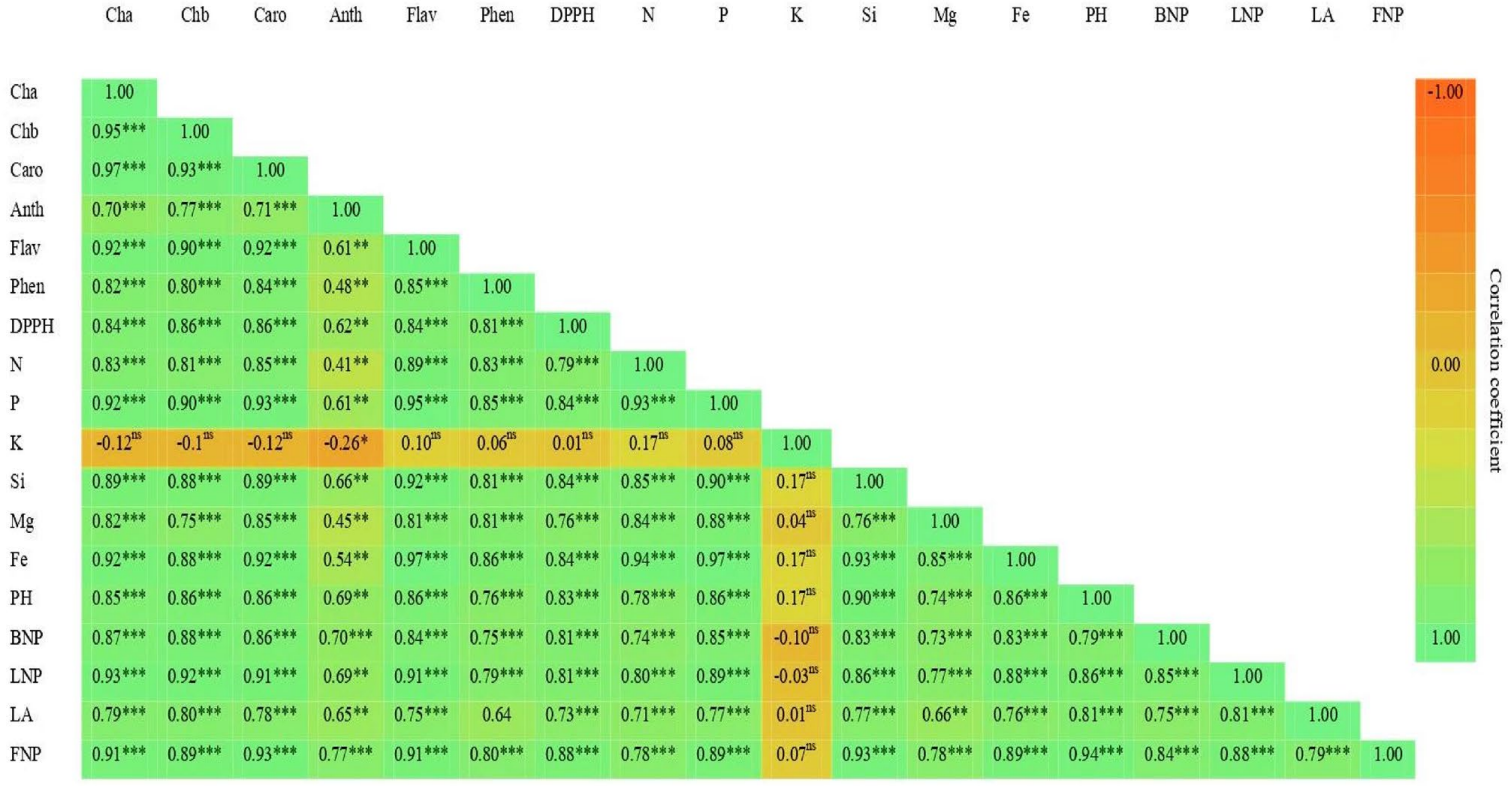
Heat map illustrates the correlation analysis between different pairs of *Brunfelsia grandiflora* traits. Cha: chlorophyll a, Chb: chlorophyll b, Caro: carotenoids, Anth: anthocyanin, Flav: flavonoids, Phen: phenolics, DPPH: total antioxidant activity, N: nitrogen, P: phosphorus, K: potassium, Si: silicon, Mg: magnesium, Fe: iron, PH: plant height, BNP: branches number plant^− 1^, LNP: leaves number plant^− 1^, LA: leaf area and FNP: flower number plant^− 1^. ns: not significant, *: significant at *P* < 0.05, **: significant at *P* < 0.01 and ***: significant at *P* < 0.001.

The correlation coefficients matrix between different biochemical and morphological parameters of *B. grandiflora* are illustrated in a heat map (Fig. 9). In this respect, the correlation coefficients between each pairs of chlorophyll a, chlorophyll b, carotenoids, anthocyanin, flavonoids, phenolics, total antioxidant activity, nitrogen, phosphorus, potassium, silicon, magnesium, iron, plant height, branches number plant^− 1^, leaves number plant^− 1^, leaf area and flower number plant^− 1^ were estimated. All possible combinations between the studied pairs of traits showed a highly significant (*p* < 0.01) positive correlation, except for the pairs involved potassium with all other traits. In this concern, there was significant (*p* < 0.05) and negative correlation between potassium and anthocyanin. Also, there were no significant correlation relationships between potassium and each of chlorophyll a, chlorophyll b, carotenoids, flavonoids, phenolics, total antioxidant activity, nitrogen, phosphorus, silicon, magnesium, iron, plant height, branches number plant^− 1^, leaves number plant^− 1^, leaf area and flower number plant^− 1^. Furthermore, the correlation between phenolics and leaf area was not significant.

## Discussion

Plant growth, development, and flowering productivity are critical for economic ornamental trees and shrubs. Si application showed positive effects on the growth and flowering of the ornamental plants^74^. Si fertilizers can enhance nutrient uptake by maximizing soil fertility and plant water absorption, while improving soil physical and chemical characteristics^46^. Additionally, Si is an abundant, practical, and non-toxic element involved in various plant functions^75^. Physiologically, Si can enhance photosynthesis at the expense of reduced transpiration, which will help with carbon build-up and nitrogen metabolism. Better leaf architecture and increased capacity for light absorption are made possible by Si absorption and subsequent formation of a double silicate layer on the leaf epidermis^76^.

The current research study was performed to examine the influence of different silicon sources (DM, PS, and SNP) on growth, flower yield as well as the chemical composition of *B. grandiflora*. Photosynthetic pigments (chlorophyll a, chlorophyll b, and carotenoids) and anthocyanin significantly were increased by application different silicon treatments. In this regard, DM, PS, and SNP enhanced chlorophyll a, chlorophyll b, and carotenoids concentrations as well as anthocyanin comparing to the check treatment. Treating plants with DM at 30% gave the greatest increase in chlorophyll a, chlorophyll b, and carotenoids in *Moringa oleifera*^77^. Foliar application of nano silicon at 75 mg L^− 1^ increased chlorophyll index (46%), stomatal conductance (34.6%), and relative water content (46.3%) in *Lathyrus sativus*^78^.

Interestingly, we observed a strong correlation between the morphological and flowering production and the secondary metabolite accumulation obtained in the leaves. For this reason, abundance of Si is essential to enhance the levels of total phenolics and flavonoids, increase the antioxidant activity, and stimulate the carbohydrates transportation to different sections of plant organs, leading to improved growth and flower production^79^. SNP at 300 mg L^− 1^ as foliar application increased total sugars, total phenolics, and total free amino acids^80^. As well, treating marigold seedlings with silicon enhanced carbohydrate contents and total flavonoids^81^.

The findings also depicted that the contents of N, P, K, Si, Mg and Fe, in response to different applications of Si, were improved comparable with control. Uses of DM improved NPK% on *Zea mays* plants compared to other treatments and the control^82^. The treatment of SNP in *Polianthes tuberosa* increased leaf P and Si contents^74^. Furthermore, treating chrysanthemum plants with PS significantly increased the content of K, P, Mg, Fe, S and Zn^83^. In another study, it was observed that spraying of Si on marigold seedlings enhanced N, P, and protein^81^. Si has a function in preventing an imbalance of nutrients during the growth and production of plants^84^. The higher nutrient content resulting from the use of Si as a fertilizer can also be attributed to the fact that it reduces the leaching of minerals such as N and K away from the root medium^85^.

Concerning the growth and flowering, it has been reported that DM increases morphological and flowering parameters in *Antirrhinum majus* plants^86^. In another study, Zaman et al. 2022^87^ revealed that applying DM increased the morphological yield of *Cicer arietinum*. Obklin et al. 2023^88^ illustrated that treated plants with DM at the rate of 5 t ha^− 1^ had a significant effect on plant height and dry weight of maize plants. It has been documented that Si increases the endogenous gibberellin formation in seedlings leading to stem elongation^89^. In addition, PS foliar application at 12 ml L^− 1^ on *Rosmarinus officinalls* showed increases in growth characteristics compared to control^90^. Moreover, using of PS at 1 to 3 g L^− 1^ as foliar application enhanced the growth and flowering traits of *Antirrhinum majus*^91^. Additionally, it should not be neglected that PS fertilizer contains K which has a stimulatory effect and crucial function in plant growth^92,93^. As for SNP, their application at 1000 mg L^− 1^ in *Rosa chinensis* had improvements of growth traits^80^. SNP spraying at 200 mg L^− 1^ and 400 mg L^− 1^ enhanced leaf fresh weight, root and bulblet dry weight, and root valium in tuberose plants^74^. In addition, using foliar application of silica nanoparticles boosted leaf area and relative chlorophyll content while improved all morphological and flowering properties of carnation plants^94^.

**Fig. 10:**
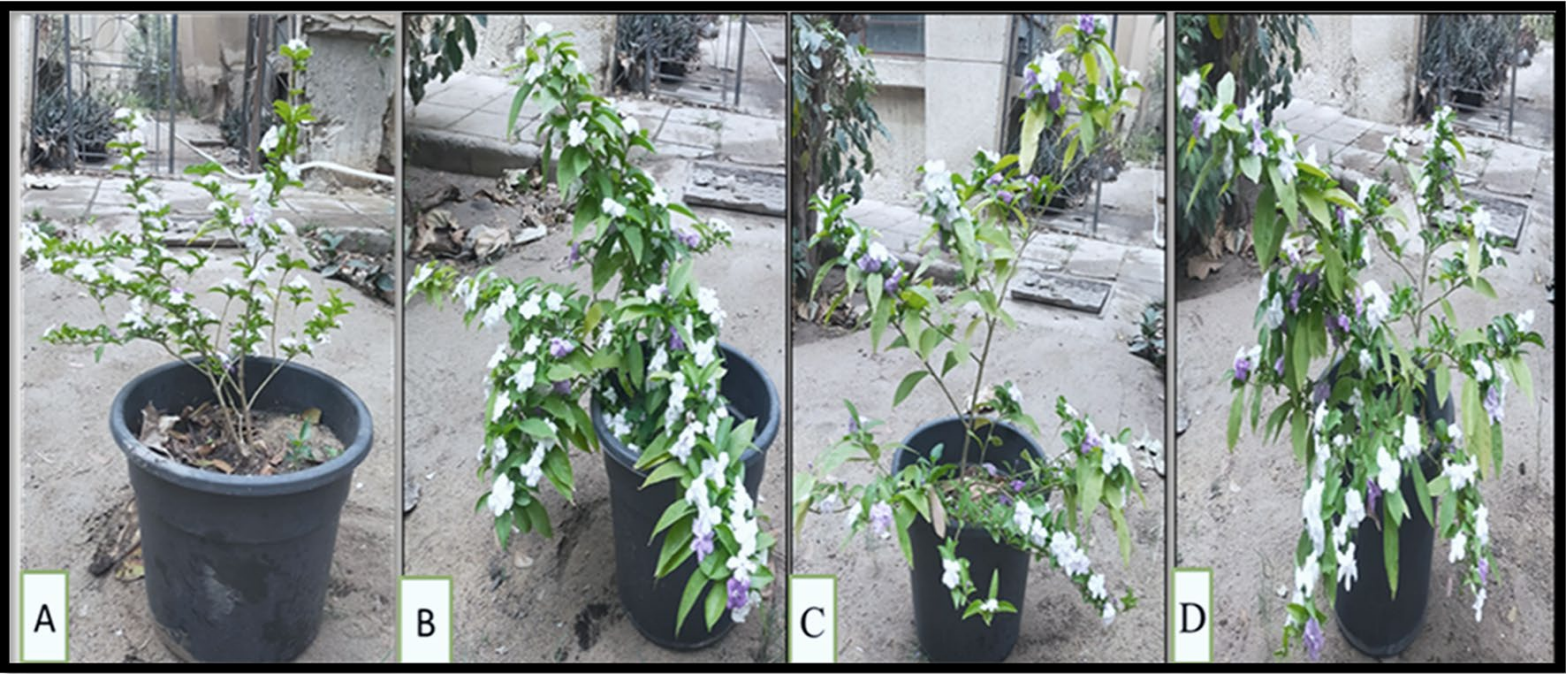
The best concentration of different silicon sources on morphology and flowering of *Brunfelsia grandiflora*, (**A**) check treatment, (**B**) diatomite 2.5%, (**C**) potassium silicate 1 g L^–1^, and (**D**) silica-nanoparticles 100 mg L^− 1^.

For economic ornamental plants, flower number is a critical parameter for beautiful aspects. More significant flowers number are essential, particularly in *B. grandiflora* because they are commonly used in landscape gardening and easily identified by the color change (violet to white) of their flowers and fragrance, and difficult to be distinguished^22^. In the current study, the flower numbers were improved significantly by the application of different silicon sources (DM, PS, and SNP) compared to the check treatment (Fig. 10). Since DM is typified by minute sized-particle with high permeability and porosity^95^, the improvements in growth and flowering of DM-treated plants are expected. Previous studies on *Polianthes tuberosa*^74^, *Antirrhinum majus*^86,91^, *Rosa chinensis*^80^, *Tagetes erecta* L^96^. , and *Lilium orientalis*^97^ cited an increase in flower yield by treating with Si as a result of increase its content in the leaves. Such findings could be ascribed to the Si’s potential impact on sugar metabolism, which could raise the concentrations of soluble carbohydrates in PS-sprayed leaves^98^. As for Si in nano from, SNP with diameters of 5–20 nm can readily penetrate the cell wall and reach the plasma membrane. They may enter through stomatal openings or at the base of trichomes, allowing foliar-applied particles to move into various tissues. Following their accumulation and translocation, SNP induce alterations in multiple cellular and physiological functions of the plant^99^. In rice, SNP have been shown to enter the xylem via active transport pathways^100^.

## Conclusions

Supplying *Brunfelsia grandiflora* plants with the appropriate form of silicon is so crucial for high flower yield and quality. In this connection, modification of plant pigments, nutrient contentment and biological molecules via application of silicon achieved substantial enhancements in growth and flowering. Herein, the tested various silicon sources, especially silica-nanoparticles and diatomite improved photosynthetic and anthocyanin pigments, total contents of phenolics, flavonoids and antioxidant activity, hence, morphological and floral traits were improved. Eventually, in *Brunfelsia grandiflora* the cultivation system, farmers have distinctive options involving silica-nanoparticles at 100 mg L^− 1^ (if available) or diatomite at 2.5% to be applied to modify the physiological and nutritional status for high yield and quality flowers.

## Data Availability

The datasets used and/or analyzed during the present investigation available from the corresponding author on reasonable request.
